# New cardiac magnetic resonance imaging modalities aid in the detection of myocardial fibrosis

**DOI:** 10.14814/phy2.13135

**Published:** 2017-03-29

**Authors:** Katherine M. Holzem

**Affiliations:** ^1^ Department of Biomedical EngineeringWashington UniversitySt. LouisMissouri63130

Heart failure (HF) is the end stage of many cardiovascular diseases, in which left ventricular (LV) function and structural remodeling are unable to meet the body's cardiac‐output demand. The burden of HF has reached an epidemic scale, with more than 20 million individuals affected worldwide (Roger 2013). Despite dramatic improvements in the clinical management of this disease, our ability to identify HF in its early stages and appropriately risk‐stratify patients for suitable treatments remains limited. As such, there has been an ongoing search for new and improved biomarkers to help us identify early disease and categorize HF into its various stages, while in tandem, advancements in imaging methods are improving our detection of structural and functional derangements resulting from various etiologies. The collective outcome of such efforts will be an improved ability to diagnose, stage, risk‐stratify, and manage HF from early subclinical manifestations to the full‐blown syndrome.

In this study, Lottonen‐Raikaslehto and colleagues (Lottonen‐Raikaslehto et al. 2017) demonstrate that the cardiac magnetic resonance (CMR) imaging modality, T1 longitudinal relaxation in the rotating frame (known as T_1*ρ*_), can be potentially used as a sensitive method to detect diffuse fibrosis in the heart, indicating that this technique may be a valuable addition to the arsenal of clinical imaging methods for cardiac diseases such as HF. Although traditional 2D echocardiography remains the initial and most frequently utilized imaging exam for HF, it continues to suffer from the major limitation of user dependence (Marwick and Schwaiger 2008; Paterson et al. 2013). In addition, accurate volume measurements, important for assessment of global ventricular size in HF, are best achieved with 3D imaging techniques, such as 3D echocardiography or CMR. Various CMR sequences and contrast‐enhanced imaging methods have more recently been adopted in clinical cardiology for their ability to provide detailed data on cardiac function, morphology, and perfusion.

More commonly used for assessments of cardiac diseases, T2‐weighted CMR has been used to show myocardial edema and inflammation. Additionally, after myocardial infarction, late‐enhancement gadolinium (LGE) CMR images can be used to identify regional fibrosis and infarct size. The combination of T2‐weighted imaging with LGE has also been useful in characterizing the majority of HF etiologies (Paterson et al. 2013). However, though LGE imaging is particularly good for localized infarct, the method correlates poorly with diffuse myocardial fibrosis based on collagen content from endomyocardial biopsies (Parsai et al. 2012). Although CMR methods to detect diffuse fibrosis are presumably most useful for nonischemic cardiomyopathy, the volume of diffuse fibrosis is twofold greater than localized scar even in ischemic cardiomyopathy.

The authors have focused on T_1*ρ*_ imaging as a method that might potentially aid in the diagnosis of cardiac failure in earlier stages of the disease, prior to the manifestations of impaired ventricular function (Lottonen‐Raikaslehto et al. 2017). Indeed, a very promising outcome of a noninvasive method to detect diffuse fibrosis within the myocardium would be in the identification of subclinical states of LV hypertrophic (LVH) remodeling. The authors have chosen a good model with which to test their hypothesis, the VEGF‐B_167_ cardiac‐specific overexpressing mouse, which demonstrates LVH‐related changes within months, but does not show LV functional compromise until after 1 year (Karpanen et al. 2008). Despite the study design, the T_1*ρ*_ CMR method, unfortunately, does not clearly demonstrate the ability to identify cardiac fibrosis preceding the onset of functional impairment in this work. The authors demonstrated a correlation between fibrosis and T_1*ρ*_ relaxation time in VEGF‐B_167_ mice at the 14‐month time point, at which they also observed a reduction in LV ejection fraction. However, the entire clinical potential of the method has not been fully explored in this study. The authors have provided foundational experiments, suggesting T_1*ρ*_ mapping correlates with the degree of fibrosis in transgene‐overexpressing mice. However, there are many additional experiments that should be performed to further illustrate the sensitivity of this method at detecting fibrosis. Various time points in the progression of LVH should be thoroughly examined in mice genetically predisposed to HF, such as the VEGF‐B_167_ mice, with T_1*ρ*_ mapping. If the method is able to detect earlier LV remodeling in predisposed mice, then other models of HF or simply aged WT mice should also be studied.

In addition to providing some interesting basic research on the ability of T_1*ρ*_ mapping to identify diffuse fibrosis in a genetic model of LVH, Lottonen‐Raikaslehto et al. (Lottonen‐Raikaslehto et al. 2017) have characterized remodeling of the VEGF‐B_167_ mouse hearts with gene expression studies and ECG measurements. LVH phenotype was demonstrated by increased expression of ANP and decreased expression of cTnT at 5, 10, and 14 months of age in VEGF‐B_167_ mice compared with WT controls. The authors have also performed valuable assessments of the ECG phenotype of these mice, demonstrating increased QRS duration and QT interval compared with WT controls. Given the importance of proarrhythmic remodeling in HF, the authors should be commended for not overlooking the ECG characteristics of their VEGF‐B_167_ animals.

Ultimately, this work is a timely addition to a growing field of research on T_1*ρ*_ CMR. Although there has been a large amount of research on T_1*ρ*_ mapping in the fields of osteoarthritis, cerebral ischemia, and hepatology, until recently, there have been few studies examining the use of T_1*ρ*_ mapping for cardiac diseases (Han et al. 2014). Previous studies have shown that T_1*ρ*_ mapping can be used for imaging acute myocardial infarction (Witschey et al. 2012; Musthafa et al. 2013), and more recently, the method has been applied to nonischemic heart disease, demonstrating that T_1*ρ*_ maps correlate with LGE positive regions in patients with hypertrophic cardiomyopathy (Wang et al. 2015). However, the basic science of cardiac T_1*ρ*_ imaging and its relationship with LV histopathology had not yet been elucidated, and Lottonen‐Raikalehto and colleagues (Lottonen‐Raikaslehto et al. 2017) have provided an important stepping‐stone toward achieving this goal.

## Conflicts of Interest

None declared.
